# Common and much less common scenarios in which botany is crucial for forensic pathologist and anthropologists: a series of eight case studies

**DOI:** 10.1007/s00414-020-02456-0

**Published:** 2020-12-19

**Authors:** Marco Caccianiga, Giulia Caccia, Debora Mazzarelli, Dominic Salsarola, Pasquale Poppa, Daniel Gaudio, Annalisa Cappella, Lorenzo Franceschetti, Stefano Tambuzzi, Lidia Maggioni, Cristina Cattaneo

**Affiliations:** 1 Dipartimento di Bioscienze, Università degli Studi di Milano, Milan, Italy; 2 Labanof (Laboratorio di Antropologia e Odontologia Forense), Sezione di Medicina Legale, Dipartimento di Scienze Biomediche per la Salute, Università degli Studi di Milano, Milan, Italy; 3 Department of Anthropology, Masaryk University, Brno, Czech Republic

**Keywords:** Forensic botany, Skeletonised human remains, Dendrochronology, Concealment locations, Murder weapon, PMI

## Abstract

It is commonly accepted that crime scene recovery and recording are key moments of any judicial inspection in which investigators must decide on the correct strategies to put into place. Complex outdoor scenarios, presenting partially or entirely skeletonised remains, can benefit more than others by the intervention of environmental specialists (forensic anthropologists, archaeologists, entomologists and botanists). These experts are capable of singling out, correctly recording and recovering environmental evidence that can lead to a more comprehensive reconstruction of a given criminal episode. If human remains are discovered in an outdoor scenario, the on-site presence of a botanist will guarantee a correct approach to the identification, recording and recovery of any botanical evidence. If an on-site botanist is not available, the operators must be capable of both the botanical evaluation of a scene and the implementation of correct botanical sampling protocols.

The following collection of unusual case histories that aim at underlining the efficacy of forensic botany will examine the determination of post mortem or the post depositional interval, evidence for a victim’s post mortem transfer, evidence for the identification of a primary crime scene and evidence for the identification of a victim’s dismemberment site. In another two cases, one, we will illustrate the important role that forensic botany played in the discrimination between botanical material used to voluntarily conceal a victim and vegetation that had grown naturally above a disposal site, whereas the other will highlight the protocols implemented for the identification of a murder weapon.

## Introduction

Forensic botany is the applied scientific discipline that regards the general study of botanical evidence in judicial investigations [1, 2] and includes many sub-disciplines, such as palynology (the study of pollen and spores), dendrochronology (the study of growth rings of tree stems and roots), lichenology (the study of lichens), mycology (the analysis and the identification of fungi) and bryology (the study of bryophytes) [3]. This array of studies can prove to be very effective in a variety of forensic scenarios, such as in determining the difference between accidental death, suicide or murder [4–6]. It can provide extremely valuable information as to the location of a burial and the interval since deposition [7–12]. Furthermore, fragments of branches, roots, leaves and seeds found at a crime scene can provide links between a discovery site and possible suspects, contributing to alibi testing and/or the determination of whether a discovery location refers to a primary or secondary crime scene [2–5, 13–16]. Finally, botanical evidence can provide useful information regarding the identification of a specific murder weapon [4]. In all these applications, the analysis of botanical traces can be carried out with the classic techniques of morphological analysis or, in some cases, with the most modern techniques of biomolecular investigation [17].

Even though over recent years, forensic botanists have been more frequently employed in outdoor crime scene investigations, however the majority of cases have only been related to well-preserved human remains [3–6, 16, 18]. As illustrated in Table 1, the majority of botanical studies of a forensic nature have been aimed at the estimation of post mortem intervals (PMI) through dendrochronological analysis or, more recently, the analysis of moss growth patterns that colonize bone surfaces [8, 19–24]. To the best of our knowledge, other cases in which forensic botany has been applied to skeletonised or partially skeletonised human remains have not, as yet, been reported in literature.

**Table 1:**
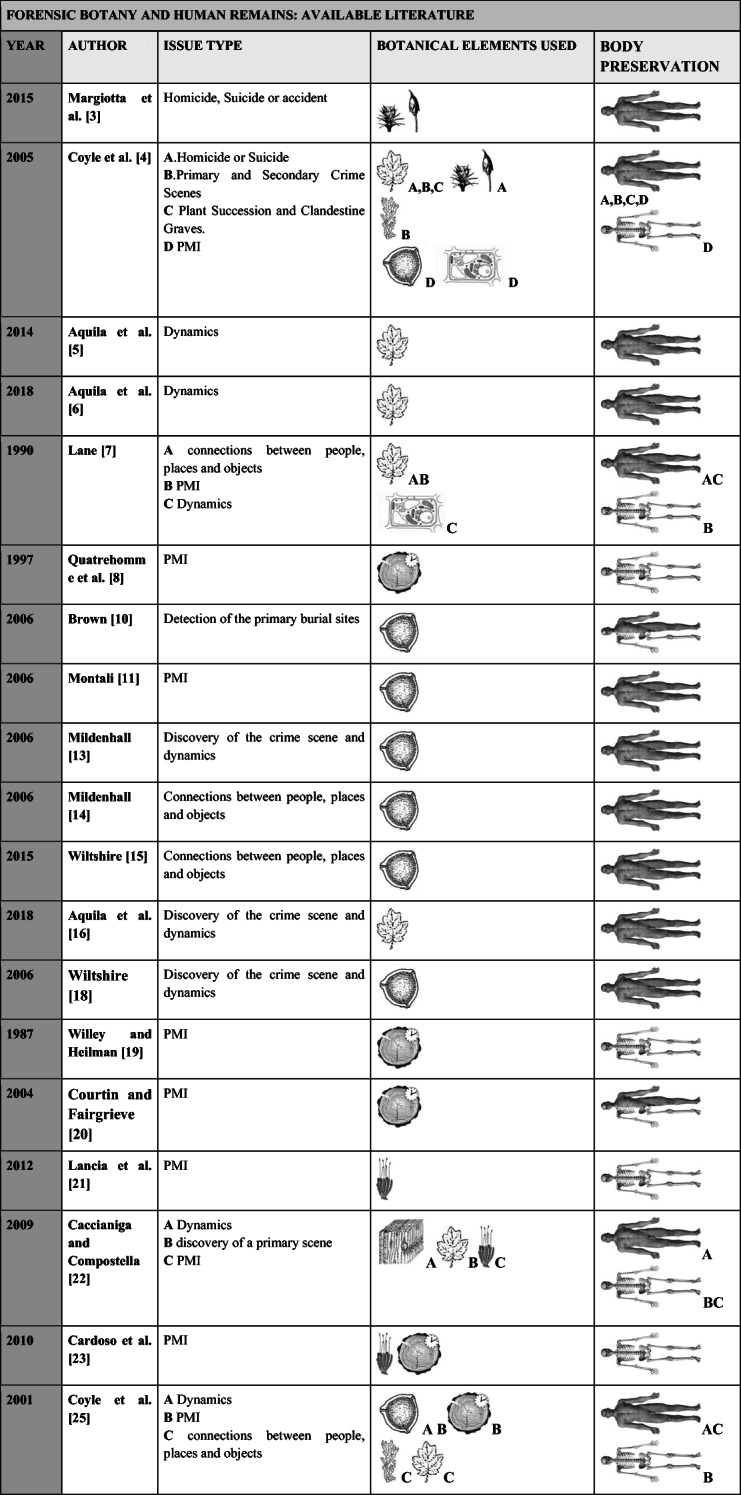
The main issues addressed, the applied techniques and the states of preservation of the examined human remains published in principle recent literature regarding forensic botany case studies

Symbols:  comparative and ecological analysis of macroremains (leaf, stems, fruits),  comparative and ecological analysis of bryophyte**,** comparative and ecological analysis of pollen**,**  comparative analysis of the microscopic morphology of wood**,**  comparative analysis of the microscopic morphology of algae**,** comparative analysis of the cell structure and other microscopic structures of leaves and fruits**,**  dendrochronology (analysis of annual growth in woody tissue) and other methods for age estimation from vegetal organs (woody and herbaceous stems),  bryophyte growth rate (analysis of annual growth of moss).  well-preserved body,  partially decomposed/ saponified, and  skeletal remains

Therefore, we are presenting eight different murder cases in which forensic botany has played an important role, providing otherwise unavailable information that contributed to the reconstruction of a specific chain of criminal events. All of the cases described involve at least partially skeletonised human remains, representing one of the most challenging and complicated scenarios that forensic pathologists will find themselves having to deal with. To the best of our knowledge, this is the first exhaustive report that presents an assembly of case studies highlighting alternative applications to forensic botany on partially or totally skeletonised human remains.

## Case 1: forensic botany and a victim’s concealment location

**Fig. 1:**
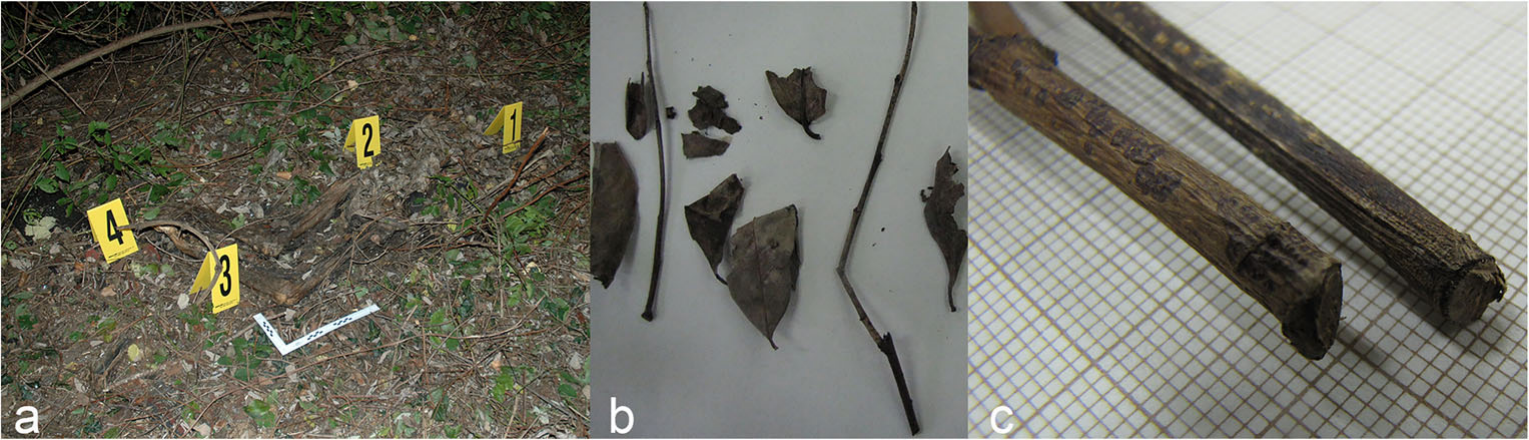
Human remains partially covered by plant components **a**, the *Prunus laurocerasus* leafs and branches collected during the on-site inspection **b** and close-up of the severed extremities of two branches **c**

In October of 2011, in a pre-Alpine valley in northern Italy, the skeletal remains of a victim were fortuitously discovered along the overgrown banks of a river, entangled in a bush, during routine maintenance work. Forensic archaeological protocols were implemented for the victim’s recovery, and evidence was positioned using a numbered grid system by a forensic anthropologist and a forensic pathologist. Above the skeletonised victim’s remains, several plant components were discovered, recorded and recovered (Fig. 1a, b). During the same inspection, the area’s spontaneous vegetation was carefully analysed, and a sample strategy was put into place for future comparison with the material that was directly associated with the remains. The remains, that were eventually identified as being those of a woman who had disappeared 3 months earlier, presented no peri-mortem trauma that could be clearly diagnostic as to the victim’s cause and manner of death. On the basis of circumstantial evidence, the woman’s husband was identified as a suspect and was subsequently charged with the murder and arrested. The vegetation that was recovered from above the victim was later analysed with the use of an episcopic microscope (Leica zoom 2000), and the leaf and stem morphology was compared to reference material and dichotomous keys. This analysis strategy provided data that led to the identification of leaves and pruned branches which recovered directly above the remains as *Prunus laurocerasus*, a shrub species not present amongst the spontaneous vegetation sampled in the discovery area (Fig.1b, c). However, a pile of severed branches and leaves discovered approximately 100 m from the discovery site, even though not pertaining to the same species as those found above the victim, led specialists to identify the general area as a clandestine dump site, typically used for the disposal of garden waste. The experts concluded that the perpetrator had hidden the woman’s body in a bush that was already growing in the area and had further concealed it by covering it with *Prunus laurocerasus* plant trimmings, recovered from a nearby fly tip. Thanks also to the support of the botanical evidence presented above, the suspect was sentenced by the Court of Appeal and subsequently by the Cassation Court (Italy’s supreme Court of Appeal) for both his wife’s murder and for the aggravated circumstance of the unlawful disposal of a dead body.

## Case 2: forensic botany and the identification of a murder weapon

**Fig. 2:**
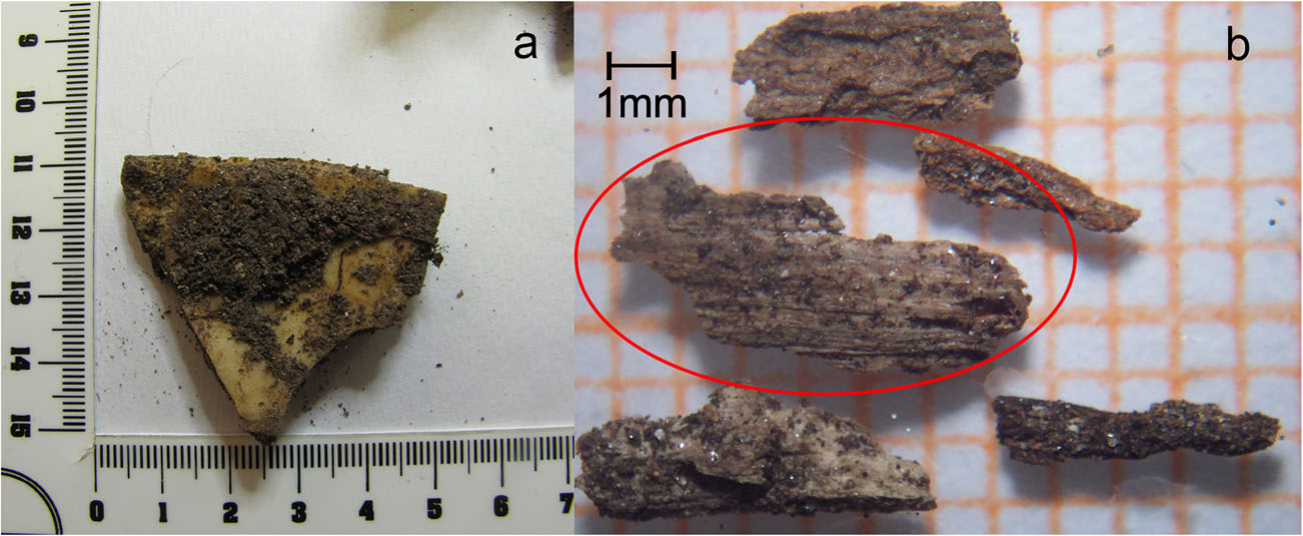
Bone fragment belonging to the frontal region of the victim’s skull **a** splinters of wood associated with the fracture in the victim’s skull; the fragment circled in red was microscopically analysed and identified as *Castanea sativa* **b**

In June 2010, skeletal human remains were found in a rugged woodland area in Canton Ticino (Switzerland). Following anthropological and genetic analysis, these were later attributed to a girl who had disappeared the previous year just a few hours after having met a young male subject. In order to thoroughly investigate the victim’s deposition and to obtain any information regarding the possible transfer of the corpse, botanical investigations were carried out. The scene was inspected by a forensic botanist who sampled and identified the discovery site’s predominant surrounding species and litter. The specimens that were directly associated with the human remains were analysed through the use of an episcopic microscope (Leica zoom 2000) and where necessary, with an optical microscope (Leitz). All of the recovered material was then photographed and identified through comparison with reference collections and dichotomous keys. All the examined specimens were consistent with the overall vegetation that constituted the woodland. Furthermore, the analysis of each of the victim’s bones revealed botanical traces that were consistent with the species found in the plant litter directly associated with each of the victim’s specific anatomical regions, thus indicating a limited movement of skeletal remains during decomposition. In particular, some wood fragments that were found embedded in a fracture line along the skull’s frontal region were considered to be relevant (Fig. 2a); however, they were found to be too small for typical microscopic analysis of the cross, radial and tangent sections and were therefore subjected to comparative analysis through episcopic and optical microscopy that provided the splinters’ diagnostic compatibility with the *Castanea sativa* (chestnut) (Fig. 2b). The anthropological analysis of the recovered cranial fragments determined the fracture to be the result of traumatic injury caused by a blunt object with a reduced striking surface. The presence of wood splinters embedded within the cranial fracture and the anthropologist’s determination of the type of injury both concurred with the use of a wooden object as the murder weapon. Even though it could not be excluded that the splinters embedded in the cranial fracture derived from plant litter, the subsequent comparative analysis with a nearby pile of chestnut blocks, probably cut from the surrounding trees did, however, provide full compatibility with the suspected murder weapon.

## Case 3: forensic botany and PMI estimation

**Fig. 3:**
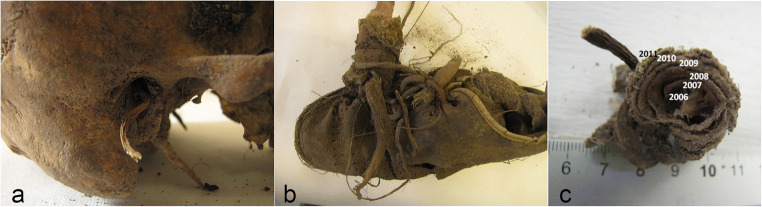
The right side of the skull with a large root penetrating the acoustic meatus **a**, the right shoe containing skeletal remains and encircled by a *Phytolacca Americana* root **b**, and close-up shot of the sampled root **c**

In July of 2011, during road maintenance work, skeletal human remains were discovered lying in an uncultivated area adjacent to a main road. During the crime scene inspection, the importance of the complex interaction between vegetal elements, in particular root systems, and the human remains became immediately apparent and called for the expertise of an on-site forensic botanist. Firstly, the specialist implemented a sampling strategy in order to carry out a comparative analysis between the local vegetation and the specimens that were subsequently recovered from the human remains. The victim’s skull was characterized by the presence of the large root that penetrated the right acoustic meatus (Fig. 3a). The left shoe, that still contained bones belonging to the victim’s foot, was deeply penetrated by a series of finer roots belonging to a bramble specimen, *Rubus* sp. In the same manner, the right shoe also containing remains of the victim’s foot presented a *Phytolacca americana* root that had developed through the shoe’s eyelets and tightly encircled the entire article (Fig. 3b). With the aim of estimating the post depositional interval, the context required an *ante quem terminus* that was estimated through the implementation of the dendrochronological analysis of several of the roots that were intrinsically associated with the skeletal remains: the roots in the acoustic meatus and associated with the victim’s vertebra were estimated at a year development, the roots in the left shoe evidenced a 3-year-old development and the roots that developed in the right shoe presented a 6-year growth (Fig. 3c).

Therefore, it was possible to determine a relatively precise *terminus post quem non,* establishing the time of the victim’s disposal to a date that was at least prior to 2006. A less certain, but probable maximum term for the victim’s disposal was also provided by botanical analysis allowing specialists to narrow down the victim’s disposal to just a few years. A thick hedge composed of *Ligustrum sp*. mixed with invasive plant species such as *Sambucus nigra* ran adjacent to the disposal site. The combined presence of the hedge, that was estimated as being a maximum of 20 years of age and the extremely invasive *Sambucus nigra,* that dated back to 1999, suggest that the hedge had not been subject to any maintenance in at least the last 10 years. By cross referencing the dendrochronological data with the assumption that any pruning or maintenance work on the hedge would have led to the discovery of the victim’s remains, it was established that the illegal disposal of the dead body must have taken place between the very end of the 1990’s (indicative term), when the *Sambucus nigra* started to grow, and 2006 (certain term), when the root systems started to develop. The estimation that the two botanical examinations provided was fully confirmed following the anthropological identification of the victim that led to the disappearance of a man in 2003. Furthermore, thanks to the botanical analysis based on the relationship between the victim’s skeletal remains and the surrounding vegetal elements, it was also confirmed that the body had been merely deposited on the area’s surface, and no attempts at the burial had been made. The lack of any botanical evidence referring to other locations led to corroborate the fact that any transfer of the victim had taken place.

## Case 4: forensic botany, the estimation of PMI and environmental dynamics

In April of 2013, a skull was discovered on a river bank in an Alpine valley in northern Italy. The forensic investigation of the site was carried out by an interdisciplinary team of specialists including both forensic anthropologists and archaeologists, who by means of forensic archaeology survey techniques, inspected and recorded the discovery site. During the surface cleaning operations, a natural depression containing numerous post cranial bones was recorded. Due to the complex interaction that takes place between human remains, soil and botanical features, it was decided that a part of the in situ crime scene should be lifted and taken to the laboratory for excavation in a controlled environment. The forensic botanist that took part in the investigation of the recovered material was asked to shed light on three distinct queries: was the recovery site the primary and only crime scene? What the estimation of the post mortem or post depositional interval was? Was there a deliberate will on behalf of a perpetrator to conceal the victim’s remains?

The material that was taken to the laboratory for analysis was recovered in such a way as to maintain the context’s integrity, and this allowed for its correct micro-excavation and the preservation of any mutual relationships between the various elements that constituted the undisturbed portion of the recovery site. The anthropological examination of the skeletal remains did not identify any peri-mortem trauma that could confirm a violent cause and manner of death.

The odontological analysis did, however, provide conclusive evidence as to the identity of the subject that corresponded to a male individual that had disappeared in the same area 16 years earlier. All of the recovered botanical elements were successfully identified. The examination revealed that the specimens were consistent with the recovery site; thus, the transfer of the subject from a primary location was, from a botanical perspective, not supported.

**Fig. 4:**
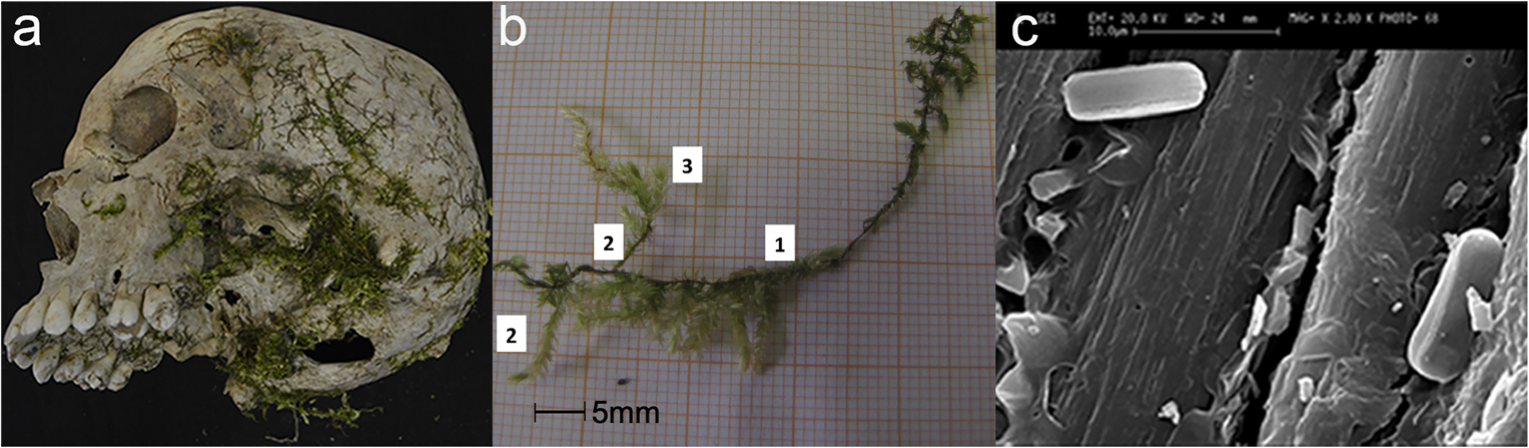
Inferior, left portion of the skull colonized by moss and, below, close-up shot of a recovered moss sample **a**, one of the recovered moss sample that colonized the left portion of the skull **b**, and SEM image of the victim’s sweater in which two diatoms are visible **c**

The second part of the botanical examination, geared towards the determination of the minimum PMI, was achieved by means of root and moss analysis. The moss specimens, that extensively colonized the deceased’s skull and clothes, were mainly identified as *Hygrohypnum luridum* (Fig. 4a)*.* The recovered moss sample consisted of three branches and considering a year’s growth for each branch and another year’s growth for decomposed branches that were still visible, and the specimen was dated to 4 years of age (Fig. 4b). This data concurred with the dendrochronological analysis performed on shrub roots intrinsically associated with the skeletal remains that had settled between 2002 and 2011. Considering the amount of time needed to colonize the subject’s remains, this interval could be marginally extended. On the basis of the *post quem non* interval, it was possible to chronologically place the victim’s death between the late 90’s and the early 2000’s.

Due on to the area’s morphology, hydrology and the fact that the thin layer of soil deposited above the remains (maximum 10 cm) presented a homogeneous distribution of living stems throughout its depth, the presence of dense vegetation, colluvial and alluvial deposits were all elements that were considered to be consistent with the progressive accumulation of soils and botanical elements throughout the post depositional interval. The presence of rounded mineral fragments recovered at the discovery site and the presence of frequent freshwater diatoms on the subject’s clothing (Fig. 4c) but not on the bone samples were highlighted by stereo electron microscopic (SEM) analysis and further confirmed the hypothesis that the discovery site was periodically affected by the flooding of a river that ran just a few meters below the deposition. This was further corroborated by available historical data regarding the river’s frequent change in water levels. The surface growth horizon was also recorded through the height of the shrub collars that were positioned just a few centimetres above the remains. For these reasons, the discovery site was considered to be that of a surface deposition that had been gradually covered by natural soil accumulation, presenting no evidence for any attempts of concealment or burial.

Botanical analysis along with anthropological and medico-legal considerations concluded that the human remains belonged to a single male subject that had decomposed in situ and had died of unknown causes between the late 90s and the early 2000s.

## Case 5: forensic botany and the confirmation of a primary crime scene

At the end of February 2011, the partially skeletonised corpse of a girl that had disappeared in the previous November was found in a field in a sparsely populated industrial area in northern Italy. The authorities carried out a large and prolonged search campaign over a wide area that also included the discovery site. Due to this, when at last the victim was discovered, presenting evident signs of violence and foul play, the search operators and the authorities suspected that the victim may have been murdered elsewhere and subsequently transferred and deposited at the discovery site. During the initial inspection, the on-site pathologist and forensic archaeologist that recovered the victim observed a deep interconnection between the human remains and plant specimens directly associated with the corpse. In the days following the victim’s recovery, a forensic botanist carried out an on-site inspection in order to implement a sampling strategy to determine the vegetation species that occupied the surrounding area, the adjacent area and the area directly beneath the profile of the victim’s deposition.

**Fig. 5:**
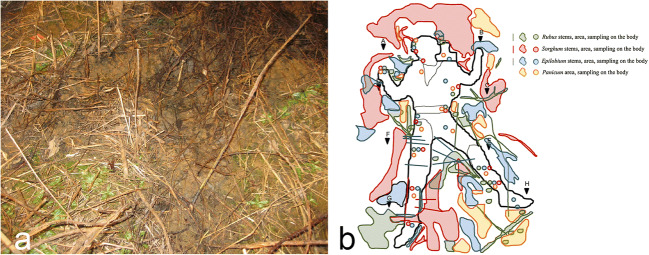
The surface beneath the victim’s body: in contrast to the adjacent areas, the seedlings of *Epilobium hirsutum* were absent. **a** Diagram illustrating the detailed analysis of the distribution of botanical elements associated with the victim and the surrounding area **b**

It subsequently emerged that the field was dominated by the presence of *Buddleja davidii*, *Rubus* sp. and numerous large size forbs such as *Epilobium hirsutum*, *Solidago gigantea* and grasses such as *Sorghum halepense* and *Panicum dichotomiflorum*. The vegetation that was clenched in the victim’s right fist was identified as *Sorghum halepense* (prevalent species), *Epilobium hirsutum* and *Rubus* sp*.* Moreover, it was noted that below the area previously occupied by the cadaver *Epilobium hirsutum* seedlings were absent, this contrasted to the adjacent areas in which they grew extensively (Fig. 5a). Finally, a *Solidago gigantea* leaf was found directly beneath the victim’s skull.

The absence of botanical evidence that could lead to other different environments than that of the discovery area along with the lack of any other environmental evidence in this sense supports the theory that the field in which the victim was found was indeed the primary crime scene, and that the victim had not undergone any post mortem transferral (Fig. 5b). On the basis of other botanical findings, the estimation of the post depositional interval was also determined and was compatible with estimations made by other specialists. In fact, by observing the distribution pattern of the *Epilobium hirsutum* seedlings that did not occupy the area directly beneath the victim’s body, it was evident that the corpse had been in the same place and position before the seedlings sprouting began. As the suitable temperatures for the germination of these seedlings was reached in that area only at the beginning of February, the minimum depositional interval could be estimated at 25–30 days prior to the victim’s discovery. In regard to the maximum depositional interval and in this case, the certain post mortem interval, the estimation was reached through the analysis of the *Solidago gigantea* leaf recovered from beneath the girl’s skull. Being particularly well preserved and well laid out, unlike those exposed to atmospheric agents which appeared crumpled and damaged, it was assumed that the protection offered by the corpse directly above it allowed for the leaf to be recorded still stretched out and well hydrated. Since *Solidago gigantea* is a late summer-autumn flowering species whose leaves and stems gradually dry out from September to November, it was concluded that the corpse had been deposited on top of the leaf from the late autumn. The determination of the chain of events and the chronological time span were perfectly consistent, not only with the date of the victim’s disappearance but also with the hypothesis that the field that had already been searched in November was to be considered as the primary murder scene.

## Case 6: forensic botany and the identification of a victim’s site of dismemberment

**Fig. 6:**
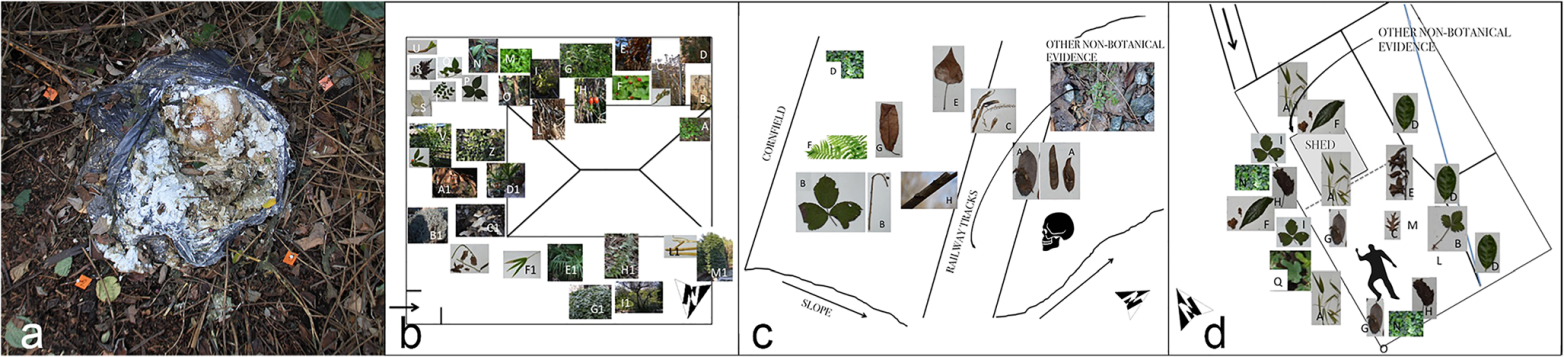
Image of the victim’s skull found in a black plastic sack **a** and diagram illustrating the three possible dismemberment locations: environment no.1 **b**, no. 2 **c** and no. 3 **d**

In September of 2017, the partially skeletised corpse of a woman who had disappeared approximately 1 month earlier was found. The victim had been decapitated and subsequently buried in a shallow grave in a suspect’s orchard. Following the search of his home, male blood-smeared clothing was recovered. Genetic analyses subsequently identified the blood as that of the missing woman. Whilst the main portion of the victim’s body was recovered during archeo-forensic investigations in the orchard, the head was missing and had evidently been transferred to an unknown secondary location. The victim’s skull, found a few days later, was closed in a black plastic sack in a wooded area not far from the suspect’s residence (Fig. 6a).

In order to identify the victim’s dismemberment site through botanical analysis, a forensic botanist carried out a series of on-site inspections in the days that followed the recovery, sampling botanical material from both the victim’s skull and each of the scenes that were suspected of being the possible site. The areas that were identified as possible places of interest were the garden of the suspect’s house (environment no. 1) (Fig. 6b), the wooded area in which the head had been recovered (environment no. 2) (Fig. 6c) and finally, the orchard where the buried body had been exhumed (environment no. 3) (Fig. 6d). On the external surface of the sack that contained the skull, botanical specimens that referred to two species were identified: *Phytolacca americana* and *Rubus* sp, whereas the inside of the sack contained a series of other botanical elements, some of which were attributed to *Robinia pseudoacacia*, *Poaceae bambusoideae* (i.e. bamboo) and *Malus domestica*. As a complete match between the botanical elements from the sack and the head’s discovery site (environment no.2) was not obtained, *Poaceae bambusoideae* (i.e. bamboo) and *Malus domestica* in fact were not present in the environment no. 2, so the specialists decided to focus on the botanical similarities between the sack’s contents and the other two locations in order to identify the environment that could have left on the remains the elements absent in the discovery site. The suspect’s garden (environment no. 1) presented only two, non-exclusive, species in common with the sack’s botanical content, *Rubus* sp*.* and *Poaceae bambusoideae*, whilst the samples gathered from the orchard (environment no. 3) provided four species in common, including the three found inside the plastic bag: *Rubus* sp*.*, *Poaceae bambusoideae*, *Robinia pseudoacacia* and *Malus domestica,* of which the latter was exclusive only to this location. On the basis of this botanical evidence, the orchard proved to be the environment that best represented all of the botanical elements associated with the victim’s skull. The orchard was therefore identified as the location in which the victim’s dismemberment probably took place, as was later confirmed by statements provided by the suspect himself.

## Case 7: forensic botany and the recovery of charred vegetal specimens

**Fig. 7:**
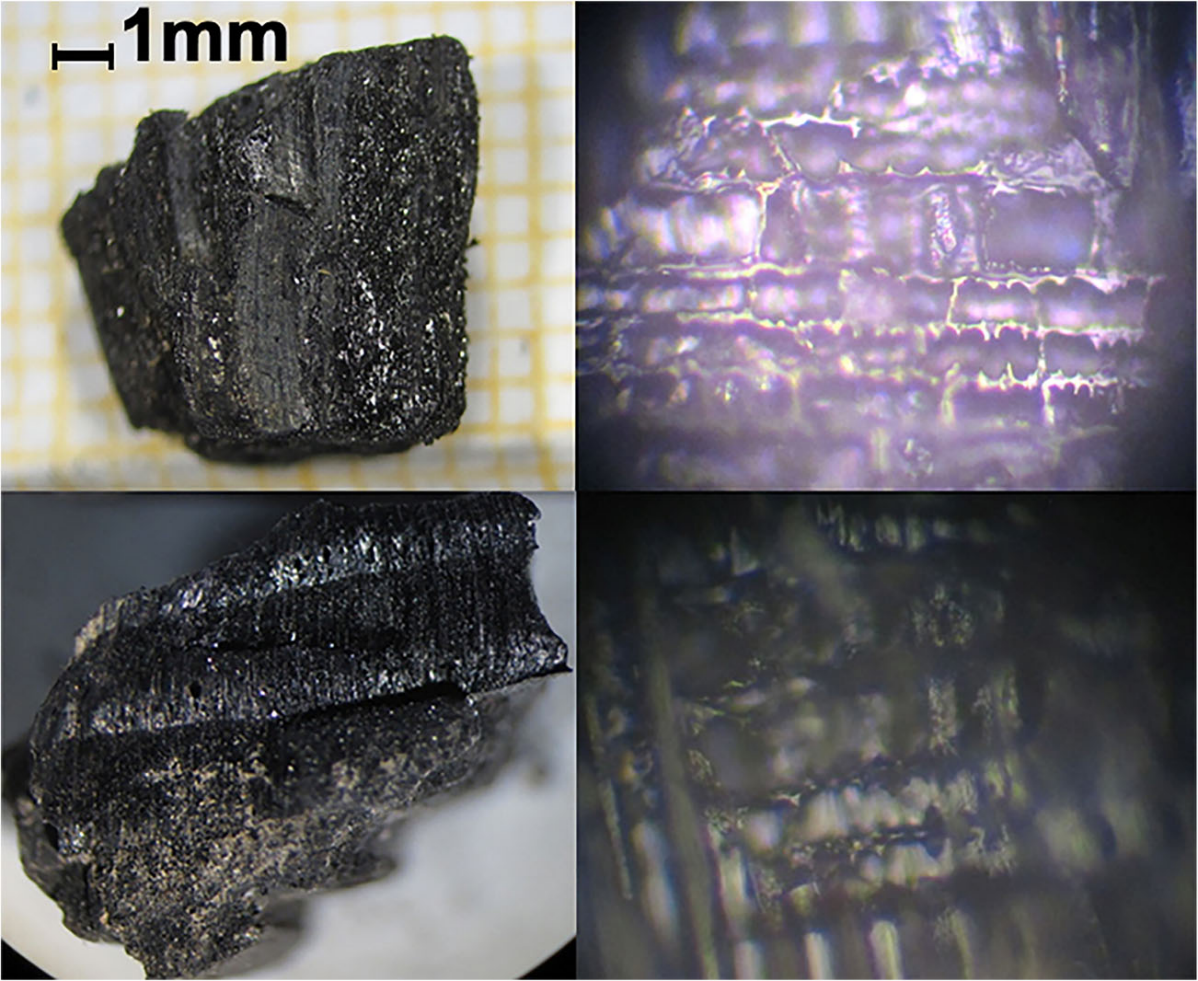
The comparison between macroscopic (left) and the × 400 microscopic (right) characteristics of a charred fragment of the pinewood found in the plastic sack (above) and a similar fragment found during the on-site inspection (below)

In November 2012, a plastic sack containing human skeletal remains belonging to a male individual was found in one of the tanks of a hydroelectric power station in the province of Gorizia in northern Italy. The victim was identified by means of DNA analysis, and it emerged that he was a missing person that had disappeared approximately 20 months earlier, around the time that his landlord had evicted him. The plastic sack contained several charred bone fragments and some botanical elements, including pieces of charred wood, whereas some of the partially burnt timber was referable to either a Mediterranean pine (*Pinus halepensis*, *P. pinaster*, *P. pinea*) or a spruce (*Picea*), and other small portions of charred branches were attributable to two distinct broad-leaved trees (*Phyladelphus* sp*.*, *Staphylea* sp*.*). Due to these findings and the fact that a few days after the victim’s disappearance, a column of smoke was seen rising from the courtyard of his old house, and a judicial on-site inspection involving a forensic botanist was undertaken. During the examination of the soil that the plastic sack contained and the subsequent sieving regime, centimetre-sized fragments of charred human bone were recovered together with charred and non-charred fragments of wood. The surviving fragments of the bone were too charred to hope for a successful DNA identification. Nevertheless, a botanical comparative analysis between the charred vegetal specimens found inside the sack and the plant species growing in the courtyard was carried out. The macroscopic characteristics of the fragments of pinewood associated with the bone fragments, such as their annular amplitudes and their carbonisation state, as well as microscopic diagnostic elements such as medullary rays and resin channels, all appeared to be the same and almost perfectly coincided with those sampled in the courtyard (Fig.7). In the same manner, the charred broad-leaved specimens that were gathered from the plastic sack also presented very similar characteristics to those gathered during the sampling campaign. Botanical analysis, therefore, revealed full consistency between the charred vegetal evidence associated with the human remains and the plant species that grew in the suspect’s courtyard, linking the victim to the site in which his corpse was very probably illegally cremated.

## Case 8: forensic botany and ingested vegetal specimens

**Fig. 8:**
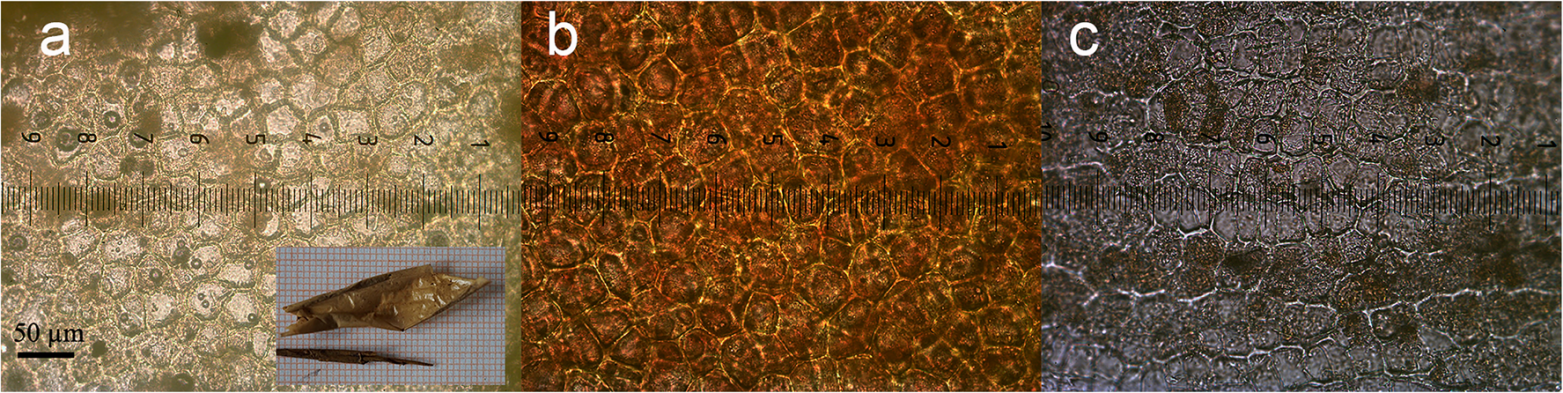
Comparison between the cellular characteristics belonging to the recovered piece of cuticle found in the victim’s colon **a**, those of a tomato **b** and a persimmon **c**

In November of 2017, a semi-closed suitcase containing partially skeletonised human remains was discovered in a woodland area in a northern Italian province. During the initial on-site inspection, the entire suitcase along with botanical samples from the surrounding area was collected by the forensic pathologist that was investigating the scene. During the subsequent autopsic examination, laminar specimens similar to cuticles with a vegetal appearance and leathery consistency were recovered from the inside of the suitcase. These were incorporated in an organic substance that was later identified as a piece of colon tract (Fig. 8a). Given the location of this discovery, incorporated in the subject’s large intestine and the morphology of the recovered elements, it was assumed that they corresponded to vegetal food remains that had been ingested by the victim some hours prior to death. Macroscopic analysis identified the fragments as belonging to a fruit’s exocarp. Microscopically, based on the characteristics of the vegetal cells, it was possible to circumscribe the specimen to either a tomato, *Solanum lycopersicum* (Fig. 8b) or a persimmon (*Diospyros kaki*) (Fig. 8c). Whilst the former could not provide particular indications in regard to the PMI, the latter due to its marked seasonality could have provided valuable information in this sense. Due to the particularly strong microscopic similarities that the two fruits present and the fact that diagnostic identification by means of bimolecular analysis resulted in being inconclusive, after consulting with a series of botanists, it was finally concluded that the fruit remnants were attributable to *Solanum lycopersicum*. Despite the difficulties encountered during the identification of the recovered evidence, the potential contribution that this kind of analysis can provide remains indisputable.

In regard to the analysis of the other botanical evidence that was recovered from the suitcase and the comparison with the samples that were gathered from the area during the on-site inspection, a significant homogeneity of specimens was recorded. This led to the conclusion that no evidence regarding any transit from previous, different locations was present. Based on the growth pattern of brambles that were absent beneath the suitcase, it was hypothesised that it had been abandoned on the discovery site’s surface before the previous growing season, probably during the second half of 2016.

## Discussion

The eight cases that have been presented in this paper involve almost entirely or at least partially skeletonised human remains in which the botanical sampling strategies were carried out with different ad hoc protocols. Regardless of the approaches implemented, all of the cases produced very clear and useful results. This was only possible due to the scrupulous attention with which the on-site experts, that were not necessarily from a botanical background, carried out the inspections, recoveries and sampling protocols. However, in both national and international contexts, this discipline is unfortunately too often underestimated due to a general lack of specifically qualified personnel who are capable of recognizing and sampling botanical evidence and to the widespread lack of culture in regard to the discipline and potential of forensic botany [3, 4, 24, 25]. It is important therefore that crime scene specialists, who are not necessarily forensic botanists, apply an interdisciplinary approach to both victim recovery and crime scene recording with the awareness of the informative power of traces of different origins; in fact, the true power lies in combining different kinds of evidence. In order for forensic botany to put to use its full potential, it is essential that botanical sampling and recovery strategies regarding both human remains and crime scenes do fulfil some minimum requirements. The exact locations at which botanical specimens have been collected must be recorded through forensic photography and 3D positioning techniques. This is aimed not only at documenting the relationships between botanical evidence and victim’s remains, but also in precisely establishing the wider botanical settings. In addition, correct recording strategies will involve written descriptions and tables, audio recorded notes, sketches and video recordings. The topographic and photographic recording is aimed at documenting the scene prior to any intervention, during operations and in the post recovery of remains and evidence. It also provides a comprehensive and extremely precise illustration of the crime scene’s three dimensional morphology, extension and position of any of the gathered botanical material. Aside from concentrating on the human remains themselves, sampling strategies involving the collection of botanical specimens belonging to the surrounding area will provide a comprehensive background documentation of a given recovery location. This should at least include the area’s predominant plant species, with particular reference to tree species that constitute the main structure and biomass of a scene’s original ecosystem. Their identification will provide overall ecological information regarding a specific site and will correspond to samples that, in all probability, will be found in direct association with a victim’s remains [2]. Finally, where possible the integral recovery and transportation of all, if not a part of the in situ deposition site to the lab can, in circumstances in which the importance of botanical information is deemed to be superior to that of other specialists, be a viable option that may guarantee a more thorough recovery and preservation of botanical data. It must however be remembered that a similar decision must be taken by the acting search and recovery manager, as this type of operation can compromise other specialists sampling and recovery strategies. As often occurs during operative activities, there are moments in which the assessment of the value of certain types of available information has to be made, and this will require decisions regarding exactly what can and cannot be sacrificed for the benefit of the investigation. It must however be underlined that the integral recovery and preservation of all contexts that have any direct or indirect physical or stratigraphic relationship to a victim will always be recovered, bagged and preserved for the further analysis.

The choice of the most suitable approach to the recording and recovery of botanical evidence can only be based on the personal experience of the expert responsible for the sampling strategy as no two cases are ever identical. In fact, no predetermined checklists or guidelines can be of assistance in the detection and recovery of all of the relevant elements that are pertinent to a specific forensic scenario [3, 4, 7]. The genesis of this problem lies in the fact that what is relevant at today’s scene may or may not be relevant tomorrow or vice versa [4]. However, some protocols such as a thorough recording strategy of the discovery site are often extremely useful and in some of the simpler cases can even be considered to be sufficient. In cases that require a later botanical inspection, once the victim has already been recovered, a further in-depth analysis can be carried out. Albeit, it should be considered that botanical elements and environments are subject to rapid alterations and are exposed to a high risk of environmental dispersion (fauna and meteorological phenomena) that can potentially modify entire distribution patterns [4].

## Conclusion

The discipline of forensic botany undoubtedly represents an important source of information in the investigation of a variety of different forensic scenarios, often providing valuable and useful information to an inquiry. In the case of skeletal or partially skeletonised human remains in which some other disciplines are simply not effective, as we have illustrated in these case studies, forensic botany can offer a great potential in the discovery of a victim’s location, the duration of its deposition and the sequence of events that may have taken place at a given scene. Forensic botany, however, can only concretely assist the forensic pathologist or investigators if the collection of botanical evidence has been carried out by forensic experts or by on-site personnel who have at least a minimum amount of training in the implementation of sampling protocols, accurate recording techniques and in the collection of background environmental data regarding a specific discovery location. Only if these preliminary on-site steps are taken correctly can the collection, identification, classification and preservation of botanical evidence be presented to an investigating body or indeed, a court of law.

Considering its potential, the discipline of forensic botany should be taken into consideration in any scenario that can potentially present botanical evidence. As outlined and demonstrated by the cases illustrated in the present paper, the information that can be provided, even if at times only circumstantial, can certainly shed light on many of the classic queries that judicial investigators advance. It is a fact that to date, botanical evidence has been increasingly presented during court proceedings and has become a widely debated subject. For this reason, the awareness of the potential that this discipline can provide in a wide variety of scenarios must increase amongst representatives of the forensic scientific community.
